# Machine Learning Approaches for Rice Seedling Growth Stages Detection

**DOI:** 10.3389/fpls.2022.914771

**Published:** 2022-06-09

**Authors:** Suiyan Tan, Jingbin Liu, Henghui Lu, Maoyang Lan, Jie Yu, Guanzhong Liao, Yuwei Wang, Zehua Li, Long Qi, Xu Ma

**Affiliations:** ^1^College of Electronic Engineering, South China Agricultural University, Guangzhou, China; ^2^College of Engineering, South China Agricultural University, Guangzhou, China; ^3^College of Mathematics and Informatics, South China Agricultural University, Guangzhou, China

**Keywords:** rice seedling, machine learning, deep learning, growth stage, histograms of oriented gradients, SVM

## Abstract

Recognizing rice seedling growth stages to timely do field operations, such as temperature control, fertilizer, irrigation, cultivation, and disease control, is of great significance of crop management, provision of standard and well-nourished seedlings for mechanical transplanting, and increase of yield. Conventionally, rice seedling growth stage is performed manually by means of visual inspection, which is not only labor-intensive and time-consuming, but also subjective and inefficient on a large-scale field. The application of machine learning algorithms on UAV images offers a high-throughput and non-invasive alternative to manual observations and its applications in agriculture and high-throughput phenotyping are increasing. This paper presented automatic approaches to detect rice seedling of three critical stages, BBCH11, BBCH12, and BBCH13. Both traditional machine learning algorithms and deep learning algorithms were investigated the discriminative ability of the three growth stages. UAV images were captured vertically downward at 3-m height from the field. A dataset consisted of images of three growth stages of rice seedlings for three cultivars, five nursing seedling densities, and different sowing dates. In the traditional machine learning algorithm, histograms of oriented gradients (HOGs) were selected as texture features and combined with the support vector machine (SVM) classifier to recognize and classify three growth stages. The best HOG-SVM model obtained the performance with 84.9, 85.9, 84.9, and 85.4% in accuracy, average precision, average recall, and F1 score, respectively. In the deep learning algorithm, the Efficientnet family and other state-of-art CNN models (VGG16, Resnet50, and Densenet121) were adopted and investigated the performance of three growth stage classifications. EfficientnetB4 achieved the best performance among other CNN models, with 99.47, 99.53, 99.39, and 99.46% in accuracy, average precision, average recall, and F1 score, respectively. Thus, the proposed method could be effective and efficient tool to detect rice seedling growth stages.

## Introduction

Rice is the most important grain crop that feeds more than half of the world’s population (Ruiz-Sánchez et al., 2010; Abid et al., 2015; Kargbo et al., 2016). It ranks first among the grain crops in China. Currently, commercial farming of rice mostly employs transplanting techniques, where seeds are sown and raised into seedlings in the nursery trays. Seedlings are later transplanted using compatible machinery (Tan et al., 2019). Healthy, disease-free, and well-nourished seedlings with uniform growth are the prerequisites for uniform field transplantation, and these seedlings must meet certain technical standards in the system of mechanical transplanting (Biswas et al., 2000; Cheng et al., 2018). Precise temperature control and proper timing of fertilizer, irrigation, cultivation, and disease control at different seedling growth stages must be considered to raise the standard seedlings. Thus, knowing the growth stages of seedling allows growers to properly time field operations to raise seedlings. Moreover, studies found out that the age of seedling at transplanting had a great impact on grain yield. Transplanting young seedling early and with high tiller production enhanced grain yield (Pasuquin et al., 2008; Ohsumi et al., 2012). However, the rice seedlings are often raised in paddy field. Environmental factors, such as changes in temperature, solar radiation, and rainfall, affect many traits that are responsible for the growth stages, including leaf photosynthesis (Makino et al., 1994; Maruyama and Nakamura, 1997), efficiency of nitrogen (N) used for leaf photosynthesis (Nagai and Makino, 2009), leaf emergence (Hiraoka et al., 1987), leaf elongation (Cutler et al., 1980), and the allocation of biomass and N to leaf (Kanno et al., 2009). Therefore, monitoring the seedling growth stage is crucial to ensure to have seedling transplant at the most suitable age. The phenology staging system of rice refers to BBCH scale which uses a decimal code to describe the growth of crops (Lancashire et al., 1991). For example, BBCH [10–19] represents seedling stages which is here the 0–9 leaves’ development. The appropriate age of rice seedling at transplant is no later than BBCH13. At present, seedling growth stage detection mainly relies on manual field inspection, which is time-consuming, labor-intensive, and inaccurate. When large-scale field involved, manual inspections become inefficient. Therefore, there is a need for a low-cost, accurate, rapid, and objective approach for rice seedling growth stage detection.

During the entire growth cycle, crops change significantly in their external morphological structures and could be observed visually, which enables us to explore new technologies to automatically observe, detect, and distinguish different critical growth stages of crops. Computer vision technology has been reported in the application of seedling quality and growth stage detection. Tong et al. (2013) developed an improved watershed segmentation for overlapping leaf images and applied it to test the crop seedling quality. Yu et al. (2013) explored the application of computer vision to automatically detect two critical growth stages of maize, including the emergence and three-leaf stage. In this study, a crop segmentation method, namely, AP-HI, was put forward to extract the plants from images. Then, the spatial distribution feature was used to judge whether the field crop had reached the emergence stage or not. Skeleton endpoint detection was used to characterize the leaf of seedling and to judge whether the field crop had reached the three-leaf stage or not. Recently, Li et al. (2021) utilized computer vision to detect rice seedling hill in the paddy field. The preferred laboratory color model along with Otsu’s method was used to extract rice seedling information, and the skeleton of the seedling hill was extracted using the thinning algorithm to effectively characterize the morphological structure of single seedling hill. Similar studies of seedling quality detection have been reported in wheat (Zhu et al., 2016), cotton (Chen et al., 2018), and rapeseed (Zhao et al., 2018).

With the rapid development of big data technology and high-performance computing, the machine learning technology has been widely used in the recent years to meet the growing demand for fast, accurate, and non-destructive applications in precision agriculture. Numerous applications of machine learning technology are reported in agricultural automation, such as yield estimation (Yang et al., 2019; Chu and Yu, 2020; Zhao et al., 2021), disease detection (Chowdhury et al., 2021; Zhang et al., 2021; Farman et al., 2022), weeds identification (Jin et al., 2021; Pandey et al., 2021), and continuous monitoring of crop status (Han et al., 2021; Taylor and Browning, 2022).

In the traditional machine learning algorithms, color, texture, and thermal features, which are extracted from RGB, multispectral and thermal images, are then fed into different machine learning algorithms, such as nearest neighbors, linear discriminant analysis, random forest, and support vector machine (SVM) to finish specific tasks. Histograms of oriented gradient (HOG) are a feature descriptor representing an image with a set of local histograms counting the occurrences of gradient orientations within a local image cell. It was successfully applied for pedestrian detection by Dalal and Triggs (2005), and the HOG descriptors significantly outperformed existing feature sets for human detection. HOG feature is widely reported in precision agriculture. Tan et al. (2018) calculated the HOG feature vectors from original color images of blueberry fruit, and then, a linear SVM classifier was trained to detect the fruit-like regions rapidly. Abouzahir et al. (2021) used the HOG to improve the performance for weed detection. In this study, HOG blocks were used as the key points to generate the visual words. A backpropagation neural network was adopted to detect weeds and classify plants for three different crop fields. This method classified plants with an accuracy of 90.4, 92.4, and 94.1% in sugar beet, carrot, and soybean fields, respectively.

The deep learning algorithms, a relatively new area of machine learning, allow computational models that are composed of multiple processing layers to learn complex data representations using artificial intelligence for image processing and data analysis (Liakos et al., 2018). One of the main advantages of deep learning algorithms is that the step of feature extraction is performed by the model itself. The performance of deep learning algorithms far exceeds that of the traditional machine learning in many applications. In fact, deep learning has been reported in the application of crop critical growth stage detection. Velumani et al. (2020) trained a convolution neural network (CNN) to identify the presence of wheat spikes in small patches acquired by a fixed RGB camera in the field. The heading date was then estimated from the dynamics of the spike presence in the patches over time. In a similar study, Bai et al. (2018) determined the arrival of the rice heading stage by the number of the spike patches detected by a CNN network. Rasti et al. (2021) investigated wheat and barley growth stage estimation by classification of proximal images using deep learning algorithm. The classification was carried out using three different machine learning approaches on an image dataset of 12 growth stages of wheat and 11 growth stages of barley. The three machine learning approaches included a 5-layer CNN, a pretrained VGG19 network, and SVM. In the seedling growth stage detection, Samiei et al. (2020) developed four deep learning models, including the multiclass CNN, 2-class CNN, CNN-LSTM, and ConvLSTM, to classify three growth stages of two species of red clover and alfalfa. The three growth stages were emergence out of the soil, cotyledon opening, and appearance of the first leaf.

Other studies addressed critical crop growth stage detection through the analysis of a height-based continuous growth curve captured over the entire growth cycle (Zhao et al., 2021). To date, several studies of crop growth stage detection have been reported. However, to our best knowledge, few studies have been reported in rice seedling growth stages detection. On the other hand, with proper sensors well equipped on, unmanned aerial vehicle (UAV) is controllable and capable of performing multiple missions. The UAV platform exhibits many advantages, such as low cost, high spatial, and temporal resolution. Moreover, the application of UAV is highly flexible, and the use of process is relatively simple. Therefore, the combination of UAV technology and machine learning algorithms allows us to detect crop growth stage in a more precise and efficient way. The objective of this study was to explore efficient and robust ways to detect three main growth stages of rice seedlings, including BBCH11, BBCH12, and BBCH13. For this purpose, an RGB camera mounted on a UAV was used to capture the images of a rice paddy field. A total of two types of machine learning algorithms were investigated: (1) For the first time, HOG feature was extracted from the rice seedling canopy images, and then, SVM was adopted to classify the seedlings into three growing stages, BBCH11, BBCH12, and BBCH13. (2) Different deep learning models were adopted to classify the three seedling growth stages. (3) The performance of the machine learning algorithms was finally evaluated and compared by proper evaluation indexes, including accuracy, average precision, average recall, and F1 score.

## Materials and Methods

**FIGURE 1:**
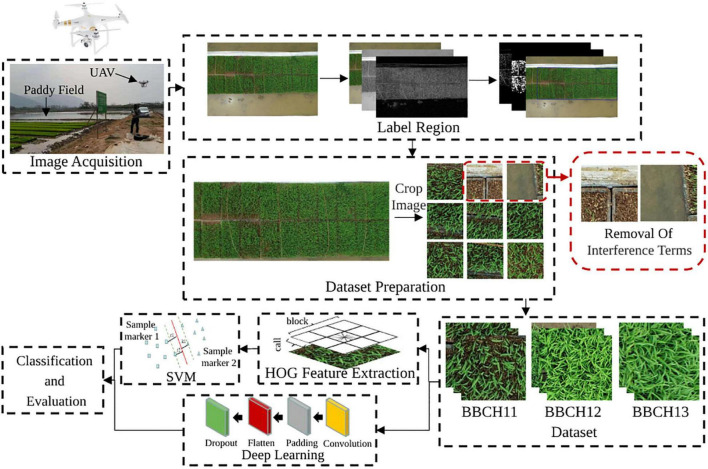
The schematic diagram of the proposed method.

In this study, the main processes of rice seedling growth stages detection, such as field data acquisition, image preprocessing, machine learning model applications, and model performance evaluations, are summarized in Figure 1. First, RGB images of rice seedling were acquired using a DJI Phantom4 RTK UAV (DJI Innovations, Shenzhen, China), and a series of image preprocessing was performed to prepare the datasets. Second, datasets created from the UAV images combined with field observations were processed through two groups of machine learning methods, namely, traditional machine learning and deep learning algorithms. Third, model performances were finally evaluated and compared, with the most desirable one(s) recommended.

### Study Sites and Field Experiments

**FIGURE 2:**
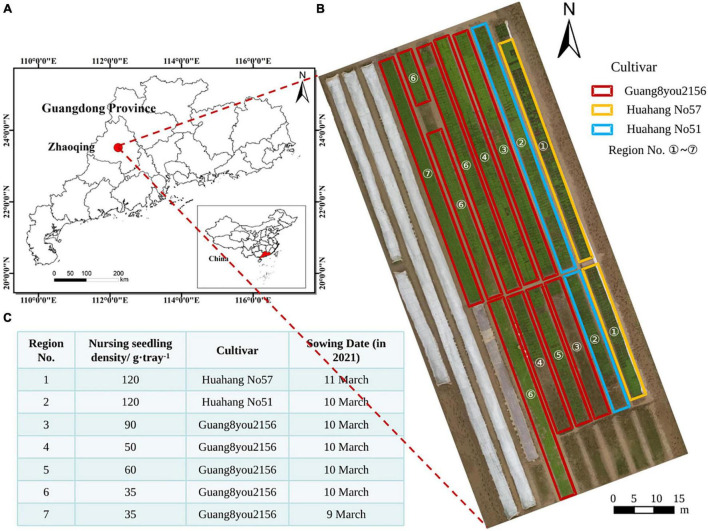
Study site and experimental design. **(A)** Location of the study site; **(B)** orthomosaic; **(C)** field tray nursing seedling experiment designs.

This study was a part of a comprehensive rice field experiments conducted at location of Research Centre Shapu, in Zhaoqing, Guangdong Province, China (23.16° N and 11.57° E). On the date 9–11 March 2021, rice seeds were sown onto the nursery trays by the 2ZSB-500 automatic precision rice seeding line. A total of three cultivars and five nursing seedling density were considered in the rice field experiment. A total of three cultivars included Huahang No. 51, Huahang No. 57, and Guang8you2156. A total of five nursing seedling densities, namely, 120 g/tray, 90 g/tray, 60 g/tray, 50 g/tray, and 35 g/tray, were adopted. After sowing seeds onto the trays, a total of 3,000 trays were classified and placed according to different sowing experiments and sowing dates. The trays were placed neatly in the paddy field. After the seedlings grew to an appropriate age, they were transplanted to a field of about 2.6 hectares for other comprehensive rice experiments. The study site and tray nursing seedling experiment design are shown in Figure 2.

### Image Acquisition and Preprocessing

#### Image Acquisition

When rice seedlings raising in the field, manual inspections by technicians were collected on 16 March, 19 March, and 24 March. In each region, the technicians sampled 100 seedlings and observed the growth stage of the seedlings. If there were more than 80 seedlings exhibited the same growth stage, this stage was recorded as the rice seedling age of the region. At the same time, the field images of this region were acquired using the DJI Phantom 4 RTK UAV with a 1-inch 20-megapixel CMOS (RGB) sensor. The images were collected with the lens shooting vertically downward, and the flight height was set to 3 m with a ground sampling distance (GSD) of 0.08 cm/pixel. The adjacent images along the flight direction overlapped on an average of one-third. The original image sizes were 5,472 × 3,642 pixels and the images were separately saved as TIFF files. On 16 March, there were less than 80 seedlings that exhibited a same growth stage in Region no 0.7 by manual inspection. Therefore, the field images of this region were excluded from the dataset. Details of RGB image acquisition and the corresponding phenological growth stages of the rice seedlings are shown in Table 1.

**TABLE 1:** Details of RGB images acquisition and the corresponding phenological growth stages of the rice seedlings.

Inspection date (2021)	16 March		19 March		24 March	
			
Region No.	Growth stage	Images acquired	Growth stage	Images acquired	Growth stage	Images acquired
1.	BBCH11	56	BBCH12	83	BBCH13	108
2.	BBCH11	46	BBCH12	60	BBCH13	85
3.	BBCH11	45	BBCH12	92	BBCH13	108
4.	BBCH11	69	BBCH12	98	BBCH13	96
5.	BBCH11	20	BBCH12	32	BBCH13	28
6.	BBCH11	84	BBCH12	120	BBCH13	102
7.	×	×	BBCH12	55	BBCH13	40

#### Image Preprocessing and Dataset Preparation

There is redundant information in the original RGB images acquired by the UAV, such as the road and the field. In addition, the large image size is not suitable for machine learning application. Hence, image preprocessing is necessary. Image preprocessing algorithm is mainly divided into three main steps (Figure 1). First, Gaussian filtering was adopted to reduce the noise after the image gray scale. Then, image enhancement is done before edge gradient detection. After that, the seedling raising regions were coarsely extracted based on the edge gradient detection, that is, RGB images of rice seedling canopy are extracted. Next, the images were cropped into different sizes, including 100 × 100 pixels, 200 × 200 pixels, 224 × 224 pixels, 300 × 300 pixels, 400 × 400 pixels, and 600 × 600 pixels. Finally, images contained redundant information were removed. The rest of the images were prepared as the datasets. Detailed information of the datasets is shown in Table 2.

**TABLE 2:** Detailed information of the datasets.

The number of images	Growth stages of the seedlings		
	
	BBCH11	BBCH12	BBCH13
Original images	320	540	567
Image of 600 × 600 pixels	1,130	3,652	4,101
Image of 400 × 400 pixels	2,814	3,545	5,739
Image of 300 × 300 pixels	4,318	5,564	4,672
Image of 200 × 200 pixels	8,083	18,994	25,842
Image of 224 × 224 pixels	12,357	27,830	25,393
Image of 100 × 100 pixels	49,840	89,333	90,759

### HOG-SVM-Based Rice Seedling Growth Stages Detection

**FIGURE 3:**
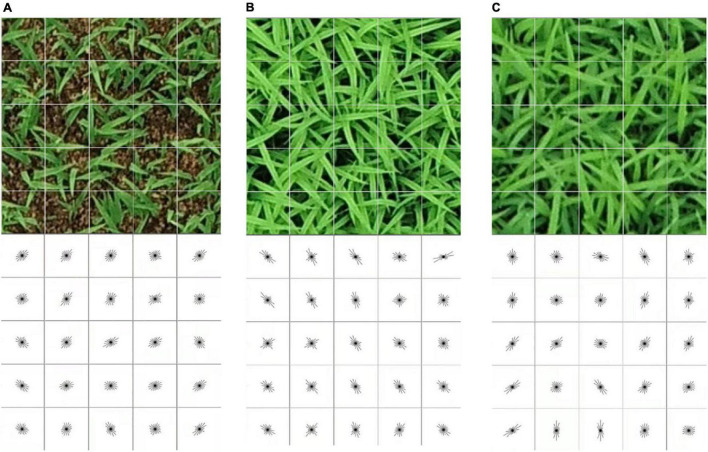
Visualization of the extracted HOG features of different seedling growth stages using a cell size of 40 × 40 and block size of 5 × 5 on image of 200 × 200 pixels. Top row, RGB images; bottom row, HOG features of rice seedlings: **(A)** BBCH11, **(B)** BBCH12, and **(C)** BBCH13.

Histograms of oriented gradient feature was used to capture and express texture features of the seedlings canopy caused by different seedling growth stages. To extract the HOG feature, the extracted images were divided into uniformly spaced non-overlapping cells of *c* × *c* pixels (Figure 3, top). The image gradient orientation of each cell was binned and aggregated into local histograms. Dalal and Triggs (2005) found that using an unsigned gradient orientation (0–180°) and 9 bins performed better than a lower number of bins and a signed gradient orientation (0–360°) with an increased number of bins (up to 18 bins). Therefore, the histogram binning was performed using the unsigned gradient orientation and 9 bins in this work. The cells were grouped into overlapping blocks of *b* × *b* cells. As such, a single cell could be included in multiple blocks. The cell histograms in each block were normalized with respect to the entire block. The HOG feature was thus comprised of all the normalized histograms of the gradient orientations (Figure 3, bottom). The cell size *c* and block size *b* were optimized through a grid search during training of the classifiers, and the block overlap was fixed to half the block size rounded up to reduce the search space.

Support vector machine has been proved to be a powerful tool for problems of classification and regression for many previous studies, and thus, it was adopted to classify the seedling images according to their growth stages based on the extracted HOG features. The images were classified into three growth stages, including the BBCH11, BBCH12, and BBCH13. Since SVMs are inherently two-class classifiers, a set of binary one-verse-one classifiers are built, which train one learning model for each pair of classes. Linear, quadratic, cubic, medium Gaussian, coarse Gaussian, and fine Gaussian functions were employed and evaluated based on their accuracy on the validation dataset.

### Deep Learning-Based Rice Seedling Growth Stages Detection

Deep learning is an important and new branch of machine learning. It originates from the artificial neural network, which learns the representation of data by constructing artificial neural network. Currently, the most widely used deep learning networks are CNNs. Efficientnet is a family of CNNs of similar architecture, which achieves more efficient results by uniformly scaling depth, width, and resolution with a scale ratio between these sets of parameters (Tan and Le, 2019). In this study, Efficientnets were adopted to classify the seedling growth stages. The performance of the proposed model was compared with the state-of-art CNN models such as VGG16, ResNet50, and DenseNet121.

#### Efficientnet

Recently, Tan and Le (2019) developed Efficientnet architectures, which were based on CNN design, and systematic model scaling technique was developed by applying a simple but effective compounded coefficient to scale up all depth, width, and resolution dimensions evenly. Tan and Le (2019) showed that the Efficientnet leads to superior performance and higher efficiency than the existing CNN methods both in terms of the number of parameters and Top1 accuracy when applied to the ImageNet dataset. Efficientnet family consists of eight models, ranging from B0 to B7. With the increase of the version, the performance of the models improves gradually, but the corresponding model size and calculation resource will not increase considerably. The main building block in Efficientnet is the mobile inverted bottleneck convolution (MBConv), which is initially introduced with MobileNetV2. The MBConv block receives two inputs, the first one is data and the second is arguments of the block. In addition, blocks consist of a layer that first expands the channels and then compresses them, thereby reducing the number of channels for the subsequent layer. A set of attributes, such as input filters, output filters, expansion rate, and compression rate, are used in the MBConv. The network parameters of EfficientnetB0 are shown in Table 3.

**TABLE 3:** Parameters of the EfficientnetB0 network.

Stage(*i*)	Operator (*F*_*i*_)	Resolution (*H*_*i*_× *W*_*i*_)	Channels (*C*_*i*_)	Layers (*L*_*i*_)
1	Conv3 × 3	224 × 224	32	1
2	MBConv1, k3 × 3	112 × 112	16	1
3	MBConv6, k3 × 3	112 × 112	24	2
4	MBConv6, k5 × 5	56 × 56	40	2
5	MBConv6, k3 × 3	28 × 28	80	3
6	MBConv6, k5 × 5	14 × 14	112	3
7	MBConv6, k5 × 5	14 × 14	192	4
8	MBConv6, k3 × 3	7 × 7	320	1
9	Conv1 × 1 & Pooling & FC	7 × 7	1,280	1

**FIGURE 4:**
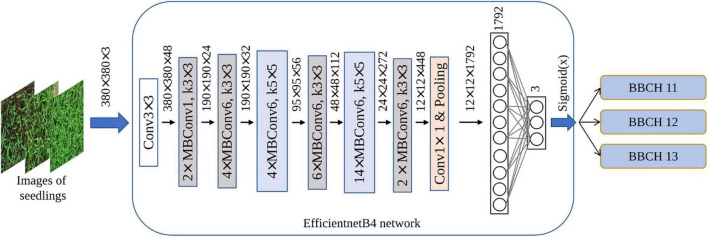
Diagram of the EfficientnetB4 used to detect rice seedling growth stages.

In this study, Efficientnet architectures were utilized to detect seedling growth stages to determine the best model. A total of two fully connected layers were added, 1,792 nodes for the inner layer and 3 nodes for the output layer (according to the number of predicted growth stages types). Figure 4 shows the diagram of the EfficientnetB4 used to detect rice seedling growth stages.

#### Other State-of-Art Convolution Neural Network Models

VGG16 presented by Simonyan and Zisserman (2015), which won the ILSVRC 2014, is a CNN architecture with approximately 138 million parameters. It consists of 5 maximum pooling layers, 13 convolution layers, 3 full connection layers, and a softmax classifier layer. Instead of having large number of hyperparameters, VGG16 always has the same convolution layers that use 3 × 3 filters with stride 1 and same padding and maximum pooling layers that use 2 × 2 filters with stride 2. All hidden layers are added with ReLU layers. After the first and second fully connected layers, the dropout technology is also used to prevent network overfitting. The input layer takes images of 224 × 224 pixels.

ResNet50 presented by He et al. (2016), which won the ILSVRC-2015 competition in 2015, is an architecture proposed to solve the problem of gradient disappearance and degradation problem. The architecture of ResNet50 is based on many stacked residual units. Residual units are used as the building blocks to build the network. These units consist of convolution and pooling layers. This architecture uses 3 × 3 filters as VGG16 and takes input images of 224 × 224 pixels.

DenseNet (Huang et al., 2017) was presented and won the best paper on CVPR2017. It encourages feature reuse and alleviates the problem of vanishing gradient. It is characterized in that DenseNet connects each layer with every other layer in a feed-forward manner, that is, the feature maps of all the previous layers are used as inputs for each layer, and their feature maps are used in all subsequent layers as inputs. This architecture has a dense connectivity pattern, therefore called a dense convolutional neural network.

### Transfer Learning

Transfer learning recycles previously trained networks using the new data to update a small part of the original weights, which makes the learning process more efficient. Given that sufficient public dataset for rice seedlings does not exist, it is difficult to obtain a satisfactory result based on the training deep learning model from scratch. Therefore, transfer learning technology (Weiss et al., 2016) was adopted in our model training. First, to obtain the pretrained network, the Efficientnets are pretrained on ImageNet, which is currently the largest image recognition dataset in the world, with 1.2 million images of 1,000 categories. Then, the seedling images are loaded into the pretrained Efficientnets. Second, the last few layers of the trained network can be removed, and two new fully connected layers are built and retrained for the growth stage classification task (Figure 4). In the transfer learning approach, using the knowledge of the network previously trained with large amounts of visual data in a new task is very advantageous in terms of saving time and achieving high accuracy compared to training the model from scratch.

### Training and Testing

In the HOG-SVM-based machine learning algorithm, 1,000 images in each growth stage, a total of 3,000 images, were randomly selected to form the basis dataset for SVM classifications. Then, the datasets in each growth stage were randomly shuffled and divided into training, validation, and test sets according to the ratio of 6:2:2. The HOG features have two hyperparameters, which were the cell size *c* ∈ {8,16,32} and the block size *b* ∈ {2,3,4,5,6}. The two hyperparameters were optimized through a grid search on the training set by training a multiclass SVM for each combination and evaluating it on the validation set. Different SVM kernels and the sizes of input image were also evaluated for each of the SVMs trained in the grid searches. In the grid search of each HOG features, the combination of the kernel function and the input image size with the best performance was selected as the optimal kernel function and input image size. Afterward, the highest accuracy on the validation set was used to select the HOG hyperparameters.

In the deep learning classification, Efficientnets are adopted to perform the classification task of rice seedling growth stage, and then, the state-of-art CNN models are considered and compared with the best performance of the Efficientnets. Table 2 formed the basis dataset for deep learning-based growth stage classification. The dataset in each growth stage was divided into training, validation, and test sets according to the ratio of 6:2:2. According to the different requirements of input image sizes of deep learning models, images of 224 × 224 pixels were prepared for the EfficientnetB0. Then, they were resized to 240 × 240 pixels and 260 × 260 pixels, which were used for EfficientnetB1 and EfficientnetB2, respectively. Images of 300 × 300 pixels were prepared for EfficientnetB3. Similarly, images of 400 × 400 pixels were resized to 380 × 380 pixels and 456 × 456 pixels and then were fed into EfficientB4 and EfficientnetB5, respectively. Images of 600 × 600 pixels were used for EfficientnetB7 and then were resized to 528 × 528 pixels and used for EfficientnetB6. Images of 224 × 224 pixels were used for VGG16, ResNet50, and DenseNet121.

Our study was conducted in Windows 10 environment (processor: Intel core i9 10920X CPU; memory: 64G; graphics card: GeForce RTX 2080Ti 11G DDR6). Python3.8 was selected for image preprocessing, whereas the feature extraction and analysis were performed in MATLAB (version 2020b, the MathWorks, Inc., Natick, Massachusetts, United States) using the Computer Vision System Toolbox 9.3 and the Classification Learner App from the Statistical and Machine Learning Toolbox 12.0. The deep learning frameworks Pytorch1.8.1 and Python3.7, in combination with Cuda10.2, were used for deep learning model training. In the experiment design and training process of deep learning models, the initial learning rate was set to 0.001, and the network batch size of the training set and validation set was set to 32. Adam optimization algorithm was selected in this work. The epoch of network model was set to 50.

### Performance Evaluation

In this study, the performance of machine learning algorithms was evaluated using four evaluation indexes of accuracy, precision, recall, and F1 score, which were given by the equations (1)-(4). The accuracy indicates the rate of correctly classified images out of all the images in a test set for a particular growth stage class, which shows the overall effectiveness of the classifier. The precision represents the proportion of images that are true positive among all images predicted to be positive. The recall represents the proportion of images predicted to be positive among the images that are true positive. The values of four evaluation indexes range from 0 to1. The higher the value is, the better the efficiency of the algorithm is.


$$a\,c\,c\,u\,r\,a\,c\,y\,\frac{\sum c\,o\,r\,r\,e\,c\,t\,l\,y\,c\,l\,a\,s\,s\,i\,f\,i\,e\,d\,i\,m\,a\,g\,e\,s}{\sum i\,m\,a\,g\,e\,s}\,\left(1\right)$$

$$p\,r\,e\,c\,i\,s\,i\,o\,n\,\left(G\,S\right)\,\frac{\sum i\,m\,a\,g\,e\,s\,w\,i\,t\,h\,G\,S\,c\,l\,a\,s\,s\,i\,f\,i\,e\,d\,a\,s\,G\,S}{\sum i\,m\,a\,g\,e\,s\,c\,l\,a\,s\,s\,i\,f\,i\,e\,d\,a\,s\,G\,S}\,\left(2\right)$$

$$r\,e\,c\,a\,l\,l\,\left(G\,S\right)\,\frac{\sum i\,m\,a\,g\,e\,s\,w\,i\,t\,h\,G\,S\,c\,l\,a\,s\,s\,i\,f\,i\,e\,d\,a\,s\,G\,S}{\sum i\,m\,a\,g\,e\,s\,w\,i\,t\,h\,G\,S}\,\left(3\right)$$

$$F_{1}\,\frac{2\,p\,r\,e\,c\,i\,s\,i\,o\,n\,r\,e\,c\,a\,l\,l}{p\,r\,e\,c\,i\,s\,i\,o\,n+r\,e\,c\,a\,l\,l}\,\left(4\right)$$

where *GS* is the growth stage: “BBCH11,” “BBCH12,” or “BBCH13.”

## Results and Analysis

### Results of HOG-SVM-Based Rice Seedling Growth Stages Detection

**FIGURE 5:**
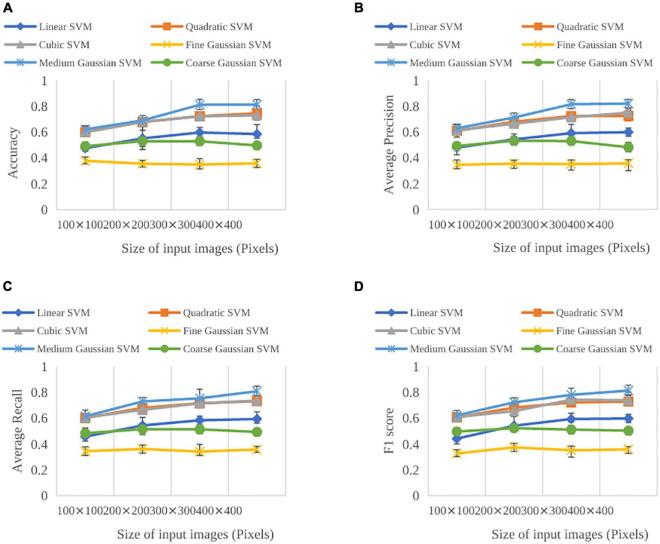
Evaluation performance on the validation sets for SVMs trained on the HOG features as a function of SVM kernels and input image sizes: **(A)** Accuracy, **(B)** average precision, **(C)** average recall, and **(D)** F1 score. Error bars show the standard deviation across the SVMs.

A total of six SVM kernels and four input image sizes were first considered. Six kernels included linear, quadratic, cubic, medium Gaussian, coarse Gaussian, and fine Gaussian whereas four input image sizes included 100 × 100 pixels, 200 × 200 pixels, 300 × 300 pixels, and 400 × 400 pixels. Figure 5 shows the performance evaluation of SVM classifiers with different kernels and input image sizes. For different kernels, medium Gaussian kernel resulted in the best in accuracy, average precision, average recall, and F1 score. The fine Gaussian kernel obtained the poorest results. Moreover, when input image size was selected as 400 × 400 pixels, the best performance was achieved, with accuracy, average precision, average recall, and F1 score of 84.91, 85.958, 84.90, and 85.43%, respectively. Therefore, the medium Gaussian kernel and image sizes of 400 × 400 pixels were chosen in the further analysis of the HOG features.

**FIGURE 6:**
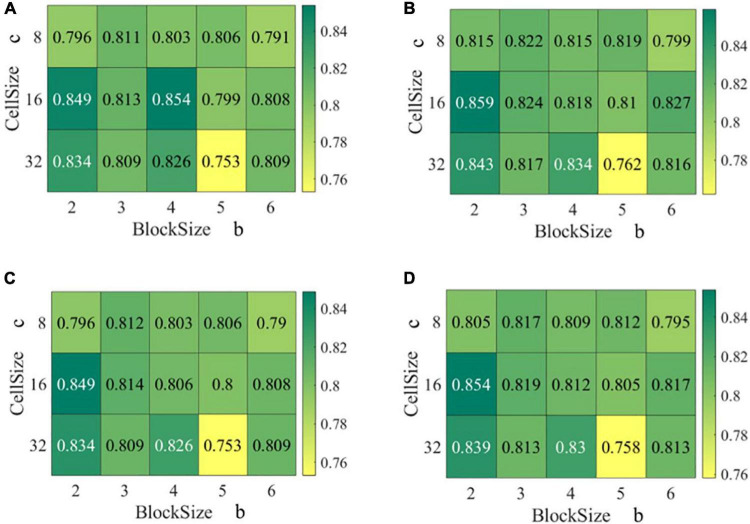
Evaluation performance on the validation sets for SVMs trained on the HOG features as a function of cell size *c* and block size *b*: **(A)** Accuracy, **(B)** average precision, **(C)** average recall, and **(D)** F1 score.

After selecting the optimal SVM kernel and input image size, the HOG feature hyperparameter grid search was performed by training individual SVM classifiers on the training set and subsequently evaluating the classifiers on the validation set and test set. Cell sizes *c* of 8, 16, and 32 pixels as well as block sizes *b* of 2, 3, 4, 5, and 6 cells were evaluated. Using the medium Gaussian kernel and the input image size of 400 × 400 pixels, the accuracy, average precision, average recall, and F1 score across the different cell size *c* and block size *b* varied from 75.3 to 85.4, 76.2 to 85.9, 75.3 to 84.9, and 75.8 to 85.4%, respectively (Figure 6). Compared with the four evaluation indexes, they showed similar trends with respect to cell size *c* or block size *b*. However, when inspected the evaluation indexes separately, they showed no clear trends with respect to cell size *c* or block size *b*.

The HOG feature with a cell size of 16 and a block size of 4 resulted in the highest accuracy of 85.4%, and the second highest accuracy of 84.9% was found in HOG feature with a cell size of 16 and block size of 2. Moreover, HOG feature with cell size of 16 and block size of 2 resulted in the highest average precision, average recall, and F1 score. Therefore, the cell size of 16 and block size of 2 were chosen as the optimal parameters.

In addition, the SVM classifier trained with the HOG feature with a cell size of 16 and a block size of 2 was evaluated on the test set. Table 4 shows the corresponding confusion matrix. A high precision (97.18%) and an adequate precision (85.34%) were found in BBCH11 and BBCH13, respectively, whereas the BBCH12 group showed an inadequate precision (75.32%). On the other hand, the recall rates in each growth stage group showed adequate performance, which achieved above 81%. Besides, BBCH12 and BBCH13 overlapped the most, which indicated that it was difficult to distinguish between BBCH12 and BBCH13.

**TABLE 4:** Confusion matrix for SVM using HOG feature with cell size of 16 and block size of 2 evaluated on the test set.

		Predicted			
		
		BBCH11	BBCH12	BBCH13	Recall
Observed	BBCH11	172	20	9	85.57%
	BBCH12	5	177	19	88.06%
	BBCH13	0	38	163	81.09%
Precision		97.18%	75.32%	85.34%	84.91%

*The lower right cell shows the accuracy.*

To further verify the robustness of the HOG-SVM model, different numbers of images were used to train the SVM model, and 1,000 images, 1,500 images, 2,000 images, and 2,500 images in each growth stage were randomly selected and formed the classification datasets. Training, validation, and test sets were divided according to the ratio of 6:2:2. Medium Gaussian kernel and HOG feature of cell size of 16 and block size of 2 were used. The performance of SVMs is shown in Table 5. The accuracy, average precision, average recall, and F1 score of different number of training images varied from 79.8 to 84.9, 80.8 to 85.9, 79.8 to 84.9, and 80.3 to 85.4%, respectively. As the number of training image increased, the performance of the SVM classifiers dropped slightly. However, the lowest F1 score was above 80%, which indicated that the overall performance was reasonably robust.

**TABLE 5:** Evaluation performance on test sets for HOG-SVM classifiers with different numbers of training images in each growth stage.

Input image size(pixels)	Number of training images in each growth stage	Accuracy	Average precision	Average recall	F1 score	Time of training(min)	Time of testing(sec)
400 × 400	1,000	84.9%	85.9%	84.9%	85.4%	3.03	12.374
400 × 400	1,500	81.0%	83.3%	81.1%	82.2%	4.16	35.741
400 × 400	2,000	81.4%	83.0%	81.4%	82.2%	7.24	52.320
400 × 400	2,500	79.8%	80.8%	79.8%	80.3%	11.6	76.156

### Results of Deep Learning-Based Rice Seedling Growth Stages Detection

#### Results of Rice Seedling Growth Stages Detection Based on Efficientnet

All Efficientnets B0-B7 were trained on the training set and then validated and tested on validation set and test set, respectively. The performance evaluations of accuracy, average precision, average recall, and F1score obtained from all Efficientnet models on validation datasets are provided in Table 6. For all eight models, classification results showed good performance. Accuracies were recorded in the range of 97.52–99.47%, whereas the average precision, average recall, and F1 score varied in the ranges of 97.54 to 99.53, 97.71 to 99.39, and 97.62 to 99.46%, respectively. The classification accuracy got better as the version of Efficientnet increased; however, there were slight decreases after EfficientnetB4. Table 6 shows that EfficientnetB4 outperformed other Efficientnet models and achieved the best values in four evaluation indexes. Table 7 shows the confusion matrix of EfficientnetB4 evaluated on the test datasets. The EfficientnetB4 showed satisfactory results. Precision and recall in each growth stage got height values, which were above 98.17%. Among them, precision in BBCH12 and recall in BBCH11 and BBCH13 obtained 100%. The classifier incorrectly recognized 3 and 10 out of 709 images (0.4 and 1.4%) of BBCH12 as BBCH11 and BBCH13, respectively. Thus, the recall rate in BBCH12 was less lower than the BBCH11 and BBCH13.

**TABLE 6:** Evaluation performance on the validation sets for EfficientnetB0-B7.

Models	Accuracy	Average precision	Average recall	F1 score	Time of training (min)	Time of test (sec)
EfficientnetB0	97.67**%**	97.54**%**	97.71**%**	97.62**%**	106.7	32
EfficientnetB1	97.52**%**	97.54**%**	97.75**%**	97.64**%**	40.9	12
EfficientnetB2	97.61**%**	97.61**%**	97.79**%**	97.70**%**	36.5	12
EfficientnetB3	98.43**%**	98.41**%**	98.58**%**	98.50**%**	50.0	15
EfficientnetB4	99.47**%**	99.53**%**	99.39**%**	99.46**%**	62.5	19
EfficientnetB5	98.78**%**	99.09**%**	99.01**%**	99.05**%**	83.2	24
EfficientnetB6	98.28**%**	98.60**%**	98.46**%**	98.53**%**	135.0	40
EfficientnetB7	98.72**%**	98.85**%**	98.74**%**	98.79**%**	220.9	66

**TABLE 7:** Confusion matrix for EfficientnetB4 evaluated on the test set.

		Predicted			
		
		BBCH11	BBCH12	BBCH13	Recall
Observed	BBCH11	563	0	0	100
	BBCH12	3	696	10	98.17%
	BBCH13	0	0	1,148	100%
Precision		99.47%	100%	99.14%	99.47%

*The lower right cell shows the accuracy.*

**FIGURE 7:**
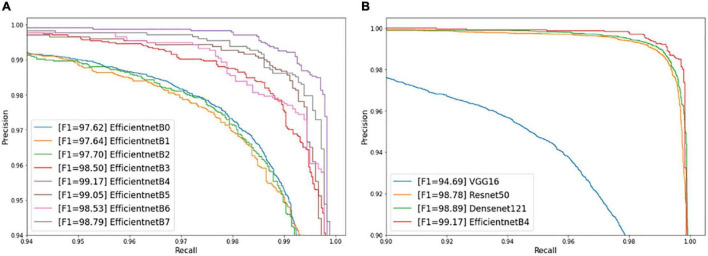
Precision–Recall curves of different deep learning models: **(A)** EfficientnetB0-B7 **(B)** State-of-art CNN models.

The precision-recall curves plot the precision rate against the recall rate. The under-area values of precision–recall curves indicate the reliability of the model from 0 to 1. The under-area values close to 1 indicates that the model can differentiate multiple classes with higher accuracy; otherwise, the smaller under-area values are, the poorer performance of the model suffers when distinguishing classes. It can be seen from Figure 7A that the under area of precision–recall curve of EfficientnetB4 is the largest, which indicates that it performs the best.

In terms of processing time of Efficientnets, as the version of Efficientnet increased from B1 to B7, the time consumption of training and test increased from 40.9 to 220.9 min, 12 to 66 s, respectively.

#### Comparison With Other State-of- Art Deep Learning Models

To verify the effectiveness of the EfficientnetB4, three other popular CNN models, VGG16, Resnet50, and Densenet121, were trained for rice seedling growth stage recognition and classification and compared with the EfficientnetB4. The mentioned CNN models were trained on the same dataset (with same hardware configuration) that were used in Efficientnet. Table 8 and Figure 7B show the performance comparison of the four models. As we can see in the table, the performance of the EfficientnetB4 achieved the best results in terms of accuracy, average precision, average recall, and F1 score. Densenet121 is close to the EfficientnetB4 model. VGG16 presented the lowest accuracy value of 94.84%. The validation results revealed that overall EfficientnetB4 performed better than the other three CNN models. In terms of processing time of popular CNNs, the time consumption of training and test of Efficientnet B4 was the lowest.

**TABLE 8:** Evaluation performance on the validation sets for different CNN models.

	Accuracy	Average precision	Average recall	F1 score	Total training time (min)	Total test time (sec)
EfficientnetB4	99.47%	99.53%	99.39%	99.46%	62.5	19
Densenet121	99.06%	98.79%	99.11%	98.95%	114.2	35
Resnet50	98.97%	98.74%	98.92%	98.83%	104.2	30
VGG16	94.84%	94.55%	94.83%	94.69%	116.7	33

## Discussion and Conclusion

### Comparison With Traditional Machine Learning Algorithms and Deep Learning Algorithms

In this paper, automatic approaches of rice seedling growth stages recognition and classification have been presented using both the traditional machine learning algorithm and deep learning algorithm. Compared with HOG-SVM-based algorithm, the performance of deep learning algorithm far exceeds that of the traditional machine learning in growth stages classification. For instance, as the best deep learning models, the EfficientnetB4 achieved the best performance, with 99.47, 99.53, 99.39, and 99.46% in accuracy, average precision, average recall, and F1 score, respectively. Meanwhile, the best HOG-SVM model obtained the performance with 84.9, 85.9, 84.9, and 85.4% in accuracy, average precision, average recall, and F1 score, respectively. In Tables 4, 7, we can notice from the confusion matrix that errors in HOG-SVM algorithm and the EfficientnetB4 mostly occur on adjacent growth stages. These are situations where even human eyes that inspect from the canopy of the seedling can have uncertainty to decide the exact growth stages from one stage to the next one. Remaining errors are low and can thus be considered as reasonable errors.

**FIGURE 8:**
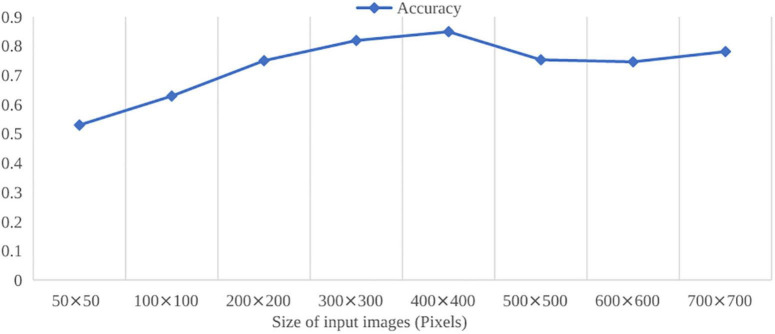
Effects of different input image size on HOG-SVM classification.

The construction of multiple layers for automatically image features learning from training data instead of complex manual feature extraction contributes to high performance of the deep learning algorithms. The phase of manual feature extraction in traditional machine learning is affected to a greater or lesser extent by many other factors and thus can sometimes result in low prediction performance. In the HOG-SVM-based rice seedling growth stage recognition, HOG features, consisting of the orientation of edges found through the computation of the image gradient, are manually selected to describe the texture feature of the seedling canopy structure. The hyperparameters of HOG, the cell size and block size, affect the number of occurrences of edges within given orientation ranges that constitute a locally spaced histogram and thus have effects on the classification performance. In addition, it can be noticed that the performance of the SVM model varies more obviously than the EfficientnetB4 does as the number of training images increases. In Tables 5, 9, as the number of training images in each growth stage increases from 1,000 to 2,500, the accuracy of HOG-SVM dropped from 84.9 to 79.8%, whereas the accuracy of EfficientnetB4 increased from 98.17 to 99.15%. Furthermore, the size of input images had effect on the performance of HOG-SVM classification. In Figure 8, the performance of classification shows the difference as the size of input images varies. The accuracy rises quickly with small input image sizes. When the input image size reaches 400 × 400 pixels, the highest value is obtained. However, the accuracy drops slightly as the image size becomes larger.

**TABLE 9:** Evaluation performance on test sets for EfficientnetB4 with different numbers of training images in each growth stage.

Model	Number of training images in each growth stage	Accuracy	Average precision	Average recall	F1 score	Time of training (min)	Time of testing (sec)
EfficientnetB4	1,000	98.17	98.20	98.17	98.18	15.8	5
EfficientnetB4	2,000	98.75	98.78	98.75	98.77	30.8	9
EfficientnetB4	2,500	99.15	99.15	99.16	99.16	45.0	13

On the other hand, the computational and processing time is a crucial aspect in machine learning algorithms. Compared to the processing time presented in Tables 5, 6, the training time of deep learning models took much longer than the HOG-SVM models. Training time of deep learning models mostly depends on the number of images used, the batch size, the learning rate, and the hardware used, among other factors. In the aspect of test time, deep learning models were faster than the HOG-SVM models. When it comes to practical application, researchers pay more attention to the test time.

In general, deep learning models exhibit satisfactory performance in rice seedling growth stage recognition. Furthermore, the datasets of rice seedling in three growth stages presented in this paper differed in genotype, sowing density, and sowing dates. A total of three cultivars and five nursing seedling densities are included in the dataset, which constitute a comprehensive seedling phenotyping. From this point of view, the traditional machine learning algorithms show reasonable discriminative ability in rice seedling growth stages.

### Limitation and Further Applications

The presented results show that the machine learning algorithms are robust on rice genotypes, sowing density, and sowing dates. However, crop phenotyping based on UAV images is also sensitive to sensor-target angles, overlap among leaves, and field conditions. In our study, all images were acquired by UAV from a vertical downward angle at a height of 3 m, producing images with similar statistical structure. To make the machine learning algorithms broadly useful across many situations, a variety of reasonable flight heights and resultant image resolutions are needed to take into account. Additionally, the minimum required image resolution (i.e., maximum flight height) that delivers quality results should be determined, because a higher flight height would allow the data to be collected more rapidly.

It has been previously reported that computer vision and machine learning techniques can help to identify the growth stages of individual seedling (Yu et al., 2013; Samiei et al., 2020). However, the issue of plant overlapping each other would decrease the detection accuracy and become a limitation. Rice is densely planted crop, and few studies have been carried out to recognize the growth stages of rice seedling. This study investigated the feasibility of developing machine learning algorithms for rice seedling growth stages detection with different canopy phenotypes. Rich information automatically learned or extracted from canopy phenotype (structural and textural information) makes it possible for the machine learning-based data analytics to achieve decision-making in a way much closer to how human brains work. It will be interesting to extend the approach to a range of crops of agricultural interest, such as oat, wheat, and sorghum, to investigate quantitatively how, by similarity in shape of different crops, the knowledge learned on rice seedlings could be transferred to others *via* transfer learning. Moreover, during the whole growth cycle, more fine growth stages, not only stages of seedlings but also stages after seedling transplantation, could also be added to extend the investigation of crop growth stage discriminative ability of machine learning algorithms.

### Conclusion

Recognizing rice seedling growth stages to timely do field operations, such as temperature control, fertilizer, irrigation, cultivation, and disease control, is of great significance of crop management, provision of standard and well-nourished seedlings for mechanical transplanting, and increase of yield. Specifically, when raising rice seedlings in paddy field, it is inefficient to manually inspect on growth stages, and environmental factors, such as rain, solar radiation have great impact on the growth stage variation. Thus, timely recognizing rice seedling growth stages become more and more important. In this study, automatic approaches using machine learning algorithms on UAV images were developed to determine three key growth stages of rice seedling, BBCH11, BBCH12, and BBCH13. In the traditional machine learning algorithm, HOG was selected as the texture feature to represent the canopy structure of the seedlings and combine with SVM classifier to recognize the growth stages. The best HOG-SVM showed reasonable discriminative ability in the classification task. Compared with the HOG-SVM algorithm, the deep learning algorithms showed outstanding performance in detection of seedling growth stages. Generally speaking, the machine learning algorithms proposed in this paper could be used to estimate the growth stages of rice seedlings in the BBCH11 to BBCH13, and they provide a basis for timely seedling supplements and subsequent crop management. Future research should include experiments employing more cultivars, different crops, and more growth stages recognition and investigate other factors to further verify and optimize the algorithms in this paper.

## Data Availability Statement

The datasets, models and code used in this paper are available at the following locations: Datasets and models: https://drive.google.com/drive/folders/1AY-ro3HID9no drk2aIcTmGgjCzTggkks?usp=sharing. Code: https://github.com/imagevision-lab/rice_seedling_growth_stages_detection.

## Author Contributions

ST: conceptualization, field data acquisition, data curation, formal analysis, investigation, methodology, software, validation, visualization, writing—original draft, writing, reviewing, and editing. JL: data curation, validation, software, and writing—original draft. HL: data curation, formal analysis, software, and visualization. ML: data curation, formal analysis, writing-original draft, and visualization. JY and GL: data curation and visualization. YW: data curation and field data acquisition. ZL and LQ: investigation, methodology, project administration, and supervision. XM: conceptualization, data curation, formal analysis, funding acquisition, investigation, methodology, project administration, resources, supervision, validation, visualization, writing, reviewing, and editing. All authors contributed to the article and approved the submitted version.

## Conflict of Interest

The authors declare that the research was conducted in the absence of any commercial or financial relationships that could be construed as a potential conflict of interest.

## Publisher’s Note

All claims expressed in this article are solely those of the authors and do not necessarily represent those of their affiliated organizations, or those of the publisher, the editors and the reviewers. Any product that may be evaluated in this article, or claim that may be made by its manufacturer, is not guaranteed or endorsed by the publisher.
